# Drug-related adverse events potentially predict the efficacy of apatinib on advanced hepatocellular carcinoma

**DOI:** 10.1186/s12876-022-02542-0

**Published:** 2022-10-31

**Authors:** Xiaoying Gu, Su Zhang, Xuejiao Yang, Tao Guan, Zhenyu Hou, Manqing Cao, Huikai Li, Ti Zhang

**Affiliations:** 1 Department of Hepatobiliary Surgery, Tianjin’s Clinical Research Center for Cancer, Key Laboratory of Cancer Prevention and Therapy, Tianjin Medical University Cancer Institute and Hospital, National Clinical Research Center for Cancer, Tianjin, 300060 China; 2 Department of Gynecologic Oncology, Tianjin’s Clinical Research Center for Cancer, Key Laboratory of Cancer Prevention and Therapy, Tianjin Medical University Cancer Institute and Hospital, National Clinical Research Center for Cancer, Tianjin, 300060 China; 3 Present Address: Department of Anesthesiology, The Central Hospital of Enshi Tujia and Miao Autonomous Prefecture, 445000 EnshiHubei, China; 4 Department of Breast Surgery, Tianjin’s Clinical Research Center for Cancer, Key Laboratory of Cancer Prevention and Therapy, Tianjin Medical University Cancer Institute and Hospital, National Clinical Research Center for Cancer, Tianjin, 300060 China; 5 Present Address: Department of Hepatic Surgery, Fudan University Shanghai Cancer Center, Shanghai Medical College, Fudan University, 200032 Shanghai, People’s Republic of China

**Keywords:** Apatinib, Anti-angiogenesis therapy, Adverse Events, Hepatocellular Carcinoma, Prognosis

## Abstract

**Background:**

Hepatocellular carcinoma (HCC) is the fourth leading cause of cancer-related deaths worldwide every year, and most HCC patients are diagnosed with advanced disease and can only receive systemic treatment. TKIs are the most important components of the systemic treatment of HCC and have both good efficacy and adverse events (AEs).

**Methods:**

This analysis included 207 patients with locally advanced unresectable or metastatic HCC who received oral treatment with apatinib. We analyzed the overall survival (OS) and progression-free survival (PFS) of patients with or without corresponding AEs to evaluate which AEs can predict the efficacy of apatinib.

**Results:**

Patients with hand-foot syndrome (HFS; *p* = 0.005), proteinuria (*p* = 0.006) and diarrhea (*p* < 0.001) had significantly better OS than those without corresponding AEs, and the appearance of HFS (*p* = 0.006) and proteinuria (*p* = 0.004) was associated with longer PFS.

**Conclusion:**

Among all the AEs induced by apatinib in the treatment of advanced HCC, proteinuria could potentially predict PFS, and diarrhea was a potential predictor of OS.

**Supplementary Information:**

The online version contains supplementary material available at 10.1186/s12876-022-02542-0.

## Key points

• Summary of the established knowledge on this subject
• Antiangiogenic therapy targeting the VEGFA/VEGFR system is an important part of the systemic treatment of HCC, but at the same time, it leads to many AEs.
• Previous studies have shown that some AEs caused by TKI treatment may be on-target toxicities of drugs, which can be used as potential predictors of efficacy.
• What are the significant and/or new findings of this study?
• Among all the AEs induced by apatinib in the treatment of advanced HCC, proteinuria can potentially predict PFS, and diarrhea is a potential predictor of OS.

## Introduction

Hepatocellular carcinoma (HCC) is the fourth leading cause of mortality due to cancer worldwide every year [1]. Over 70% of HCC patients are diagnosed with advanced disease for which resection or transplantation is not suitable [2]. For these patients, systemic treatment can reduce tumor burden and prolong survival time [3, 4]. Antiangiogenic therapy is an important part of the systemic treatment of HCC [5]. Angiogenesis is mainly regulated by vascular endothelial growth factor A (VEGFA) and its receptors (VEGFRs, mainly VEGFR2), which play a key role in the tumorigenesis and development of HCC [6]. Receptor tyrosine kinase inhibitors (TKIs) can block the binding of VEGFR2 to VEGFA, thereby exerting an antitumor effect by inhibiting angiogenesis in cancer patients [6]. Currently, several TKIs, including sorafenib, lenvatinib, regorafenib and cabozantinib, are recommended for HCC according to guidelines for treating HCC [1], and the 2020 ACSO annual meeting symposium has published that apatinib significantly improves OS and PFS in Chinese patients with pretreated advanced HCC [7].

Our previous studies have shown that apatinib has better anticancer efficacy, but at the same time, it leads to many AEs [8, 9]. The most common AEs included hypertension, hand-foot syndrome (HFS), proteinuria and fatigue, and the most common grade 3/4 AEs included proteinuria, hematological toxicity and liver function abnormalities [9]. Previous studies have shown that AEs caused by TKI treatment, such as hypertension, HFS, hypothyroidism, and proteinuria, may be on-target toxicities of drugs [10, 11]. The definition of on-target toxicities is that TKIs act on normal tissues in addition to tumors, inhibiting tyrosine kinases that can regulate tumor cell survival or proliferation and lead to the development of AEs, the occurrence of which can therefore serve as potential predictors of the efficacy of TKIs [10, 11]. Our previous studies have confirmed that hypertension is a potential predictor of the efficacy of apatinib on advanced HCC. Patients who developed hypertension after apatinib treatment had significantly better OS and PFS than those without hypertension [9].

To further explore whether other AEs after apatinib treatment can predict its efficacy, we conducted a comprehensive analysis of all AEs except for hypertension. We present the following article/case in accordance with the STROBE reporting checklist.

## Patients and methods

### Patients

This study included 207 advanced HCC patients receiving oral treatment with apatinib from December 2015 to September 2020 at Tianjin Medical University Cancer Institute and Hospital.

The inclusion criteria were as follows: ≥ 18 years old, ECOG PS score 0–2; clinically proven advanced HCC, ≥ 1 measurable lesions as defined by RECIST 1.1; BCLC stage B or C, Child–Pugh class A or B; previous HCC systemic therapy ≤ 1, life expectancy ≥ 12 weeks, bilirubin ≤ 3 mg/dl, AST and ALT ≤ 5 times the upper limit of normal value, serum creatinine ≤ 3.0 mg/dl or creatinine clearance ≥ 40 mL/min, urine protein ≤ 1 + , urine protein analysis ≥ 2 + , urine protein < 1000 mg/24 h, absolute neutrophil count ≥ 1.0*10^9/L, hemoglobin ≥ 10 g/dL, platelet ≥ 50*10^9/L; international normalization ratio ≤ 1.5, partial thromboplastin time ≤ 5 s above ULN.

The exclusion criteria were as follows: systemic anticancer therapy, local therapy or surgery within 28 days prior to entry into the study; ascites that were difficult to control; brain metastases with clinical signs or meningeal carcinogenesis; bleeding of esophageal or gastric varices within 3 months prior to the study; acute hepatitis; presence of progressive central nervous system disease; clinically significant bleeding or thrombotic events within 4 weeks prior to study registration; Child–Pugh class C.

This study was approved by the Medical Ethics Committee of the Tianjin Medical University Cancer Institute and Hospital (reference number bc2019090). The ethical review board considered that it was not necessary to obtain informed consent from the participants because this retrospective study anonymously processed all data.

### Treatments

The initial dose of oral apatinib was 500 mg/day or 250 mg/day. The three regimens of apatinib dose adjustment included 500 mg/day, 250 mg/day, and 250 mg/2 days. The dose of apatinib was reduced when grade 3/4 drug-related AEs occurred, and for patients who tolerated apatinib well, it was recommended to increase the dose. Treatment was discontinued when patients experienced unacceptable AEs, radiological progression defined by RECIST 1.1, or death.

### Follow-up

Baseline assessment and tumor screening were performed within 21 days prior to apatinib treatment. Baseline and assessments were performed every 8–12 weeks, including physical examination, vital signs, ECOG PS assessment, electrocardiogram, and clinical and laboratory tests (AFP, liver function, and renal function). AEs were classified and ranked according to the National Cancer Institute General Terminology Standard (NCI-CTCAE v 4.0). A computed tomography or magnetic resonance imaging scan was performed every 8 weeks, and the tumor response was assessed according to RECIST 1.1.

### Statistical analysis

We performed propensity score matching (PSM) to reduce the impact of potential confounding factors and biases. A multivariate logistic regression model including sex, age, performance status, Child–Pugh class (A or B), AFP level (AFP > 400 μg/L or AFP ≤ 400 μg/L), BCLC stage (B or C), initial apatinib dose (250 mg/day or 500 mg/day), previous history of hepatitis (no hepatitis, hepatitis B, hepatitis C), prior treatment-TACE (yes or no), vascular invasion (yes or no), and extrahepatic metastasis (yes or no) was used to calculate the propensity scores. Following the calculation result of propensity scores, the patients were matched using 1:1 nearest neighbor matching with a caliper distance set at 0.03. Before and after matching, the log-rank test was performed to compare the PFS and OS of groups with or without corresponding AEs. Univariate and multivariate analyses were performed using the Cox proportional hazard model to evaluate independent factors affecting OS and PFS. Kaplan–Meier survival analysis was performed to generate survival curves, progression-free survival curves and summary statistics. A Cox proportional hazards model was used to estimate the HR and 95% CI. A value of *p* < 0.05 was considered to be statistically significant. All statistical analyses in this study were performed with SPSS version 25.0 (IBM Corporation, Armonk, NY, USA).

## Results

### Patient characteristics

The baseline and disease characteristics of the included patients are shown in Table 1, and the AEs based on apatinib treatment are summarized in Table 2. Only one patient subsequently received a combination therapy consisting of anti-programmed death-1 monoclonal antibody and regorafenib, and two patients were switched to other TKIs. The date of the last follow-up was September 30, 2020, and the median follow-up was 29.6 months. For all patients, the median OS was 13.7 m (95% CI, 12.1–15.4), and the median PFS was 7.8 m (95% CI, 6.7–8.9).

**Table 1:** Patient Baseline and Disease Characteristics (Total Patients, *N* = 207)

Variables	All Patients (%)
Age	
< 60 years	122 (58.9%)
≥ 60 years	85 (41.1%)
Sex	
Male	179 (86.5%)
Female	28 (13.5%)
ECOG PS	
0	99 (47.8%)
1	108 (52.2%)
Child–Pugh	
A	152 (73.4%)
B	55 (26.6%)
BCLC	
B	41 (19.8%)
C	166 (80.2%)
AFP	
< 400 μg/L	115 (55.6%)
≥ 400 μg/L	92 (44.4%)
Initial dose	
250 mg/d	192 (92.8%)
500 mg/d	15 (7.2%)
Metastasis	
MVI	100 (48.3%)
EHS	112 (54.1%)
Lung	19 (9.2%)
Bone	21 (10.1%)
Lymph node	79 (38.2%)
Other parts	25 (12.1%)
Hepatitis	
None	38 (18.4%)
Hepatitis B	160 (77.3%)
Hepatitis C	6 (2.9%)
Hepatitis B + C	3 (1.4%)
Prior Treatment	
Surgery	72 (34.8%)
TACE + TAE	153 (73.9%)
RF	25 (12.1%)
Radiotherapy	8 (3.9%)
Biotherapy	4 (1.9%)
Other targeted therapies	6 (2.9%)

*Abbreviations*: *AFP* Alpha-fetoprotein, *BCLC* Barcelona Clinic Liver Cancer, *ECOG PS* Eastern Cooperative Oncology Group performance status score, *EHS* Extrahepatic spread, *MVI* Macrovascular invasion, *RF* Radiofrequency ablation, *TACE* Transcatheter arterial chemoembolization, *TAE* Transcatheter arterial embolization

**Table 2:** Adverse Events Profile Based on Apatinib Treatment (Total Patients, *N* = 207)

Adverse Events	Any Grade, No. (%)	Grade 3 or 4, No. (%)
All Adverse Events	188 (90.8%)	78 (37.7%)
Hypertension	85 (41.1%)	1 (0.5%)
Hand and foot syndrome	81 (39.1%)	10 (4.8%)
Fatigue	78 (37.7%)	6 (2.9%)
Abnormal liver function	65 (31.4%)	14 (6.8%)
Hematological toxicity	57 (27.5%)	22 (10.6%)
Anorexia	56 (27.1%)	12 (5.8%)
Proteinuria	50 (24.2%)	25 (12.1%)
Diarrhea	47 (22.7%)	2 (1.0%)
Vomiting	32 (15.5%)	6 (2.9%)
Hoarse voice	30 (14.5%)	2 (1.0%)
Dry mouth	27 (13.0%)	0
Ascites	19 (9.2%)	6 (2.9%)
Abdominal pain	11 (5.3%)	0

### Survival analysis

**Fig. 1:**
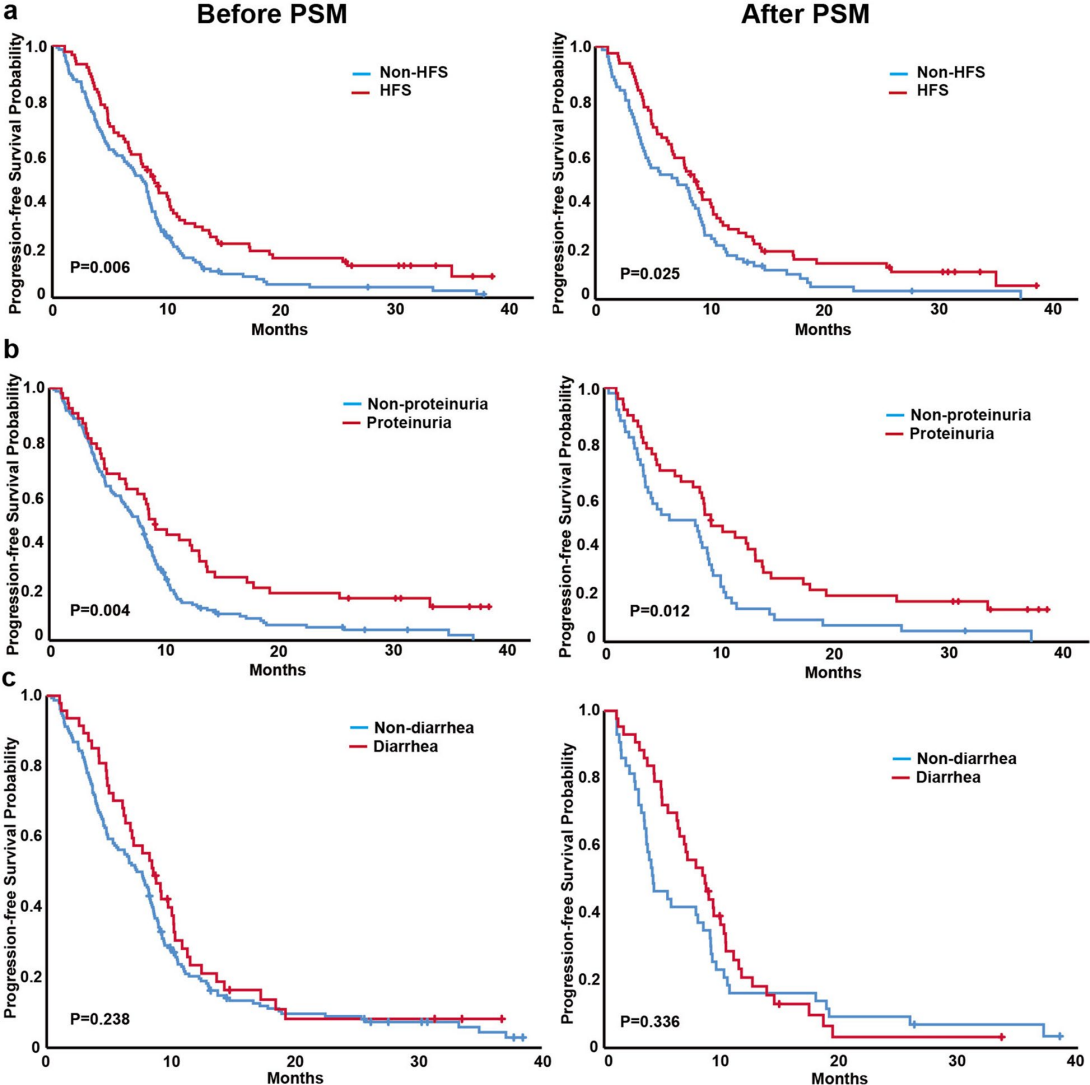
OS of patients with and without HFS, proteinuria and diarrhea before and after PSM

The analyses of all AEs that occurred after apatinib treatment except for hypertension showed that patients with HFS, proteinuria, or diarrhea had significantly better median OS than patients without corresponding AEs. The baseline characteristics of patients with and without corresponding AE before and after PSM are shown in Tables S1, 2 and 3. Before PSM, the median OS of the HFS group and non-HFS group after treatment were 20.3 m (95% CI 13.5–27.1) and 12.5 m (95% CI 11.0–13.9, *p* = 0.005), respectively (Fig. 1a); the median OS of the proteinuria group and the nonproteinuria group were 23.1 m (95% CI 11.4–34.8) and 13.0 m (95% CI 11.9–14.2, *p* = 0.006) (Fig. 1b); and that of the diarrhea group and the nondiarrhea group were 25.5 m (95% CI 15.4–35.7) and 12.5 m (95% CI 10.9–14.0, *p* < 0.001) (Fig. 1c). Similar results were obtained after PSM: the median OS of the groups with and without HFS after apatinib treatment were 18.1 m (95% CI 9.8–26.4) and 12.6 m (95% CI 10.2–14.9, *p* = 0.005) (Fig. 1a), that of the proteinuria group and the nonproteinuria group were 23.1 m (95% CI 10.4–35.8) and 8.9 m (95% CI 4.6–13.3, *p* < 0.001) (Fig. 1b); for patients with and without diarrhea, the median OS were 24.3 m (95% CI 14.6–34.1) and 8.9 m (95% CI 6.3–11.6, *p* < 0.001), respectively (Fig. 1c).

**Fig. 2:**
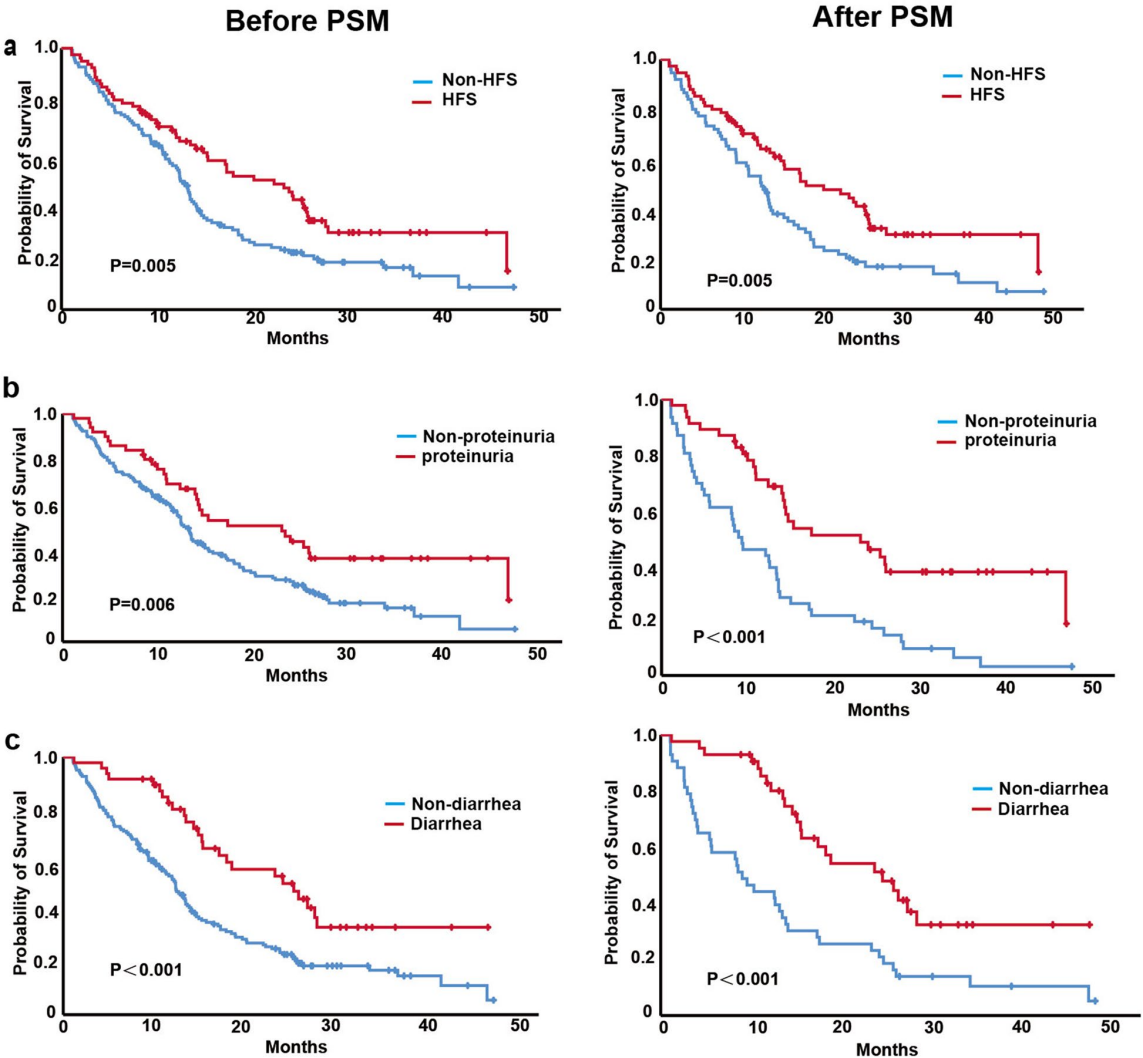
PFS of patients with and without HFS, proteinuria and diarrhea before and after PSM

The appearance of HFS and proteinuria after apatinib treatment were associated with longer PFS. For the HFS group and non-HFS group, the median PFS was 8.6 m (95% CI 7.1–10.1) and 7.1 m (95% CI 5.5–8.8, *p* = 0.006), respectively (Fig. 2a). In the proteinuria group and nonproteinuria group, the median PFS was 8.7 m (95% CI 7.6–9.8) and 7.2 m (95% CI 5.9–8.5, *p* = 0.004), respectively (Fig. 2b). After PSM, the median PFS of the HFS group and non-HFS group was 8.3 m (95% CI 6.4–10.2) and 5.7 m (95% CI 2.3–9.0, *p* = 0.025), respectively (Fig. 2a), and that of the proteinuria group and nonproteinuria group was 8.7 m (95% CI 6.8–10.7) and 5.0 m (95% CI 0.5–9.4, *p* = 0.012), respectively (Fig. 2b). There was no significant difference in PFS for patients with and without diarrhea before or after PSM (Fig. 2c).

We also analyzed the impact of the grade of these AEs on the prediction of prognosis and found no significant differences.

### Univariate and multivariate analyses

Subsequent univariate and multivariate analyses yielded results consistent with our previous study on hypertension potentially predicting the PFS and OS of advanced HCC patients [9]. Additionally, among all the AEs after apatinib treatment except for hypertension, diarrhea (HR 0.544, 95% CI 0.349–0.847; *p* = 0.007) was an independent factor potentially affecting OS (Table 3), and proteinuria (HR 0.681, 95% CI 0.470–0.988; *p* = 0.043) was a potential predictor of PFS (Table 4).

**Table 3:** Univariate and Multivariate Analyses of Variables Affecting OS in Patients (Total Patients, *N* = 207)

Variables	Univariate HR (95%CI)	*P* value	Multivariate HR (95%CI)	*P* value
Sex (male vs. female)	1.044 (0.664–1.644)	0.851		
Age (≥ 60 years vs. < 60)	0.880 (0.633–1.224)	0.447		
Initial dose				
500 mg	1.041 (0.576–1.882)	0.895		
ECOG PS 1	1.173 (0.850–1.620)	0.332		
AFP > 400	1.128 (0.819–1.555)	0.461		
Child–Pugh B	2.036 (1.444–2.873)	0.000	2.198 (1.549–3.121)	0.000
BCLC C	1.589 (1.025–2.462)	0.038	1.673 (1.078–2.596)	0.022
Hepatitis				
Hepatitis B	1.078 (0.705–1.650)	0.729		
Hepatitis C	0.684 (0.207–2.262)	0.533		
Metastasis				
MVI	1.510 (1.094–2.084)	0.012		
EHS	1.025 (0.741–1.418)	0.881		
Bone	1.206 (0.744–1.954)	0.448		
Lung	1.011 (0.601–1.701)	0.968		
Lymph node	0.951 (0.683–1.326)	0.769		
Other parts	1.408 (0.887–2.235)	0.146		
AEs				
Hypertension	0.496 (0.351–0.701)	0.000	0.652 (0.444–0.957)	0.029
HFS	0.617 (0.439–0.867)	0.005		
Proteinuria	0.568 (0.379–0.852)	0.006		
Diarrhea	0.461 (0.300–0.710)	0.000	0.544 (0.349–0.847)	0.007

*Abbreviations*: *AFP* Alpha-fetoprotein, *AEs* Adverse events, *BCLC* Barcelona Clinic Liver Cancer, *ECOG PS* Eastern Cooperative Oncology Group performance status score, *EHS* Extrahepatic spread, *HR* Hazard ratio, *HFS* Hand-foot syndrome, *MVI* Macrovascular invasion, *OS* Overall survival, *PFS* Progression-free survival, *95% CI* 95% confidence interval

**Table 4:** Univariate and Multivariate Analyses of Variables Affecting PFS in Patients (Total Patients, *N* = 207)

Variables	Univariate HR (95%CI)	*P* value	Multivariate HR (95%CI)	*P* value
Sex (male vs. female)	1.012 (0.672–1.526)	0.953		
Age (≥ 60 years vs. < 60)	0.863 (0.644–1.156)	0.322		
Initial dose				
500 mg	0.676 (0.376–1.216)	0.191		
ECOG PS 1	0.952 (0.714–1.269)	0.736		
AFP > 400	1.280 (0.960–1.707)	0.093		
Child–Pugh B	1.265 (0.919–1.743)	0.150		
BCLC C	1.385 (0.950–2.019)	0.091		
Hepatitis				
Hepatitis B	1.102 (0.758–1.604)	0.611		
Hepatitis C	1.079 (0.420–2.770)	0.875		
Metastasis				
MVI	1.255 (0.941–1.673)	0.122		
EHS	1.046 (0.784–1.396)	0.760		
Bone	1.312 (0.833–2.068)	0.242		
Lung	1.421 (0.883–2.286)	0.147		
Lymph node	0.870 (0.647–1.169)	0.356		
Other parts	1.301 (0.840–2.015)	0.238		
AEs				
Hypertension	0.515 (0.381–0.695)	0.000	0.561601 (0.410–0.768)	0.000
Proteinuria	0.601 (0.423–0.853)	0.004	0.681601 (0.470–0.988)	0.043
HFS	0.658601 (0.488–0.889)	0.006		
Diarrhea	0.812601 (0.574–1.149)	0.239		

*Abbreviations*: *AFP* Alpha-fetoprotein, *AEs* Adverse events, *BCLC* Barcelona Clinic Liver Cancer, *ECOG PS* Eastern Cooperative Oncology Group performance status score, *EHS* Extrahepatic spread, *HR* Hazard ratio, *HFS* Hand-foot syndrome, *MVI* macrovascular invasion, *PFS* Progression-free survival, *95% CI* 95% confidence interval

## Discussion

Previous studies have shown that hypertension, HFS, hypothyroidism, and proteinuria caused by TKI treatment can be used as potential predictors of efficacy. Our previous study confirmed that the development of hypertension predicted better efficacy of apatinib in advanced HCC. The median OS, PFS and TTP of the hypertension group were significantly longer than those of the nonhypertension group. In this study, we retrospectively analyzed all AEs except for hypertension after treatment with apatinib in 207 patients with advanced HCC to assess the possibility of other AEs predicting the efficacy of apatinib.

This study confirmed that in all AEs induced by apatinib treatment, in addition to hypertension, proteinuria was a potential predictor of prognosis. Patients with advanced HCC who experienced proteinuria after taking apatinib had significantly better PFS than those without proteinuria, indicating that these patients had a longer duration of sensitivity to apatinib and a later development of resistance to apatinib. Previous studies have identified proteinuria as an on-target toxicity of TKIs, and in the preliminary study of TKI-induced proteinuria, the VEGFA/VEGFR system was considered to play a key role [10–12]. These may explain the correlation between PFS and the development of proteinuria. The OS of these patients was also significantly better than that of those without proteinuria after apatinib treatment, which may be related to their longer PFS. However, it cannot be neglected that during the administration of VEGF inhibitors, serious drug-related AEs can lead to dose reduction or even the disruption of treatment, which is more obvious in proteinuria [13, 14]. Dose reduction will affect the efficacy of antiangiogenic drugs, but there is currently no effective intervention for proteinuria induced by VEGF inhibitors [15], which makes the mechanism of proteinuria an urgent problem to be solved.

Inconsistent with previous studies, our study found that diarrhea occurring with apatinib treatment was a potential predictor of OS. However, diarrhea was only positively correlated with prolonged OS, and there was no significant difference in PFS between the diarrhea group and the nondiarrhea group. These results indicated that the development of diarrhea after medication may have nothing to do with the efficacy of apatinib treatment and therefore cannot reflect the sensitivity of HCC patients to apatinib. This AE may not be due to on-target toxicity of apatinib. These patients had longer OS, which may result from other reasons. The same result was found in lenvatinib therapy on thyroid cancer, diarrhea was associated with OS in multivariate analyses but not related to PFS [16]. We speculated that HCC patients with diarrhea after apatinib treatment may benefit from changes in the intestinal flora. Previous studies mentioned that VEGFR inhibitors may lead to intestinal bacterial overgrowth and diarrhea by inhibiting c-KIT [17]. Changes in the stool microbiota were indeed found in patients with diarrhea after using VEGFR inhibitors to treat metastatic renal cell carcinoma (mRCC). For patients with diarrhea, their stool microbiota presented higher levels of Bacteroides spp. and lower levels of Prevotella spp [18]. Transplantation of fecal microbiota from healthy donors to mRCC patients with diarrhea after taking sunitinib and pazotinib significantly alleviated diarrhea [19]. These results suggest that there may be a correlation between the diarrhea induced by apatinib and the changes in gut microbiota. Currently, an increasing number of studies have focused on the function of the gut microbiota in tumorigenesis and cancer therapy and have found that changes in the gut microbiota might affect systemic antitumor immunity [20]. In liver cancer, the gut microbiota may suppress tumors by modulating HCC immunosurveillance [21]. These results further indicate that the prolonged OS of HCC patients treated with apatinib may benefit from the influence of altered gut microbiota on the immune system. However, which kind of microbiota changes are beneficial to the prognosis of cancer patients needs to be further explored. At the same time, these results also show the feasibility of alleviating diarrhea while improving the prognosis of patients by changing the gut microbiota of cancer patients through adjuvant drugs (antibiotics or probiotics). It has been found that the use of antibiotics covering Bacteroides spp improved the PFS of mRCC patients receiving VEGFR inhibitors [22]. Although diarrhea correlated with VEGFR inhibitor treatment is mainly mild to moderate [9, 16, 23–26], considering the high incidence of diarrhea and the fact that patients usually take medication for a long time, even low-grade diarrhea may affect the quality of life; therefore, diarrhea needs to be treated as soon as possible [27]. However, changes in the gut microbiota may affect the prognosis, which may require clinicians to be more cautious when choosing antidiarrheals.

This study had limitations. First, hypertension, proteinuria, HFS, and diarrhea induced by apatinib in the treatment of advanced HCC patients were all associated with OS and PFS. When patients experience two or more of these AEs at the same time, they may have longer OS and PFS than patients with only one AE. However, this study had a small sample size which could not be effectively grouped, such that the impact of multiple AEs occurring simultaneously in the same patient on OS and PFS could not be analyzed. Second, this study is a single-center, single-arm, retrospective study, and the results cannot explain the relationship between the occurrence of AEs and prognosis in the overall population, which requires a multicenter study for verification.

This study confirmed that among all the AEs induced by apatinib in the treatment of HCC, proteinuria can potentially predict better PFS, diarrhea is a potential predictor of OS, and hypertension is a potential predictor of both PFS and OS.

## Supplementary Information

**Additional file 1:** **Table S1.** Baseline characteristics of patients before and after PSM (Non-HFS vs. HFS). **Table S2.** Baseline characteristics of patients before and after PSM (Non-Proteinuria vs. Proteinuria). **Table S3.** Baseline characteristics of patients before and after PSM (Non-diarrhea vs. Diarrhea)

## Data Availability

The datasets used during this study can be obtained from the corresponding author on reasonable request.

## Abbreviations

TKI: Tyrosine kinase inhibitor
OS: Overall survival
PFS: Progression-free survival
PSM: Propensity score-matched
AFP: Alpha-fetoprotein
BCLC: Barcelona Clinic Liver Cancer
TACE: Transcatheter arterial chemoembolization
ECOG PS: Eastern Cooperative Oncology Group performance status score
AE: Adverse event
95% CI: 95% Confidence interval
HR: Hazard ratio
mRCC: Metastatic renal cell carcinoma
HCC: Hepatocellular carcinoma
MVI: Macrovascular invasion
EHS: Extrahepatic spread
TAE: Transcatheter arterial embolization
RF: Radiofrequency ablation
HFS: Hand-foot syndrome
VEGF: Vascular endothelial growth factor
TTP: Time to progression

## References

[1] Gordan JD, Kennedy EB, Abou-Alfa GK (2020). Systemic Therapy for Advanced Hepatocellular Carcinoma: ASCO Guideline. J Clin Oncol.

[2] Machairas N, Tsilimigras DI, Pawlik TM (2021). State-of-the-art surgery for hepatocellular carcinoma. Langenbeck's archives of surgery.

[3] Cainap C, Qin S, Huang WT (2015). Linifanib versus Sorafenib in patients with advanced hepatocellular carcinoma: results of a randomized phase III trial. J Clin Oncol.

[4] Zhang T, Merle P, Wang H (2021). Combination therapy for advanced hepatocellular carcinoma: do we see the light at the end of the tunnel?. Hepatobiliary Surg Nutr.

[5] Llovet JM, Montal R, Sia D (2018). Molecular therapies and precision medicine for hepatocellular carcinoma. Nature reviews Clinical oncology.

[6] Jayson GC, Kerbel R, Ellis LM (2016). Antiangiogenic therapy in oncology: current status and future directions. The Lancet.

[7] Li Q, Qin S, Gu S (2020). Apatinib as second-line therapy in Chinese patients with advanced hepatocellular carcinoma: A randomized, placebo-controlled, double-blind, phase III study. J Clin Oncol.

[8] Kong Y, Sun L, Hou Z (2017). Apatinib is effective for treatment of advanced hepatocellular carcinoma. Oncotarget.

[9] Yang X, Hou Z, Zhu K (2020). Drug-Related Hypertension Associated with the Efficacy of Apatinib on Hepatocellular Carcinoma. Cancer Manag Res.

[10] Shah DR, Shah RR, Morganroth J (2013). Tyrosine kinase inhibitors: their on-target toxicities as potential indicators of efficacy. Drug safety.

[11] Dienstmann R, Brana I, Rodon J (2011). Toxicity as a biomarker of efficacy of molecular targeted therapies: focus on EGFR and VEGF inhibiting anticancer drugs. Oncologist.

[12] Gu X, Zhang S, Zhang T (2021). Abnormal Crosstalk between Endothelial Cells and Podocytes Mediates Tyrosine Kinase Inhibitor (TKI)-Induced Nephrotoxicity. Cells.

[13] Sato J, Satouchi M, Itoh S (2020). Lenvatinib in patients with advanced or metastatic thymic carcinoma (REMORA): a multicentre, phase 2 trial. Lancet Oncol.

[14] Nakagawa K, Garon EB, Seto T (2019). Ramucirumab plus erlotinib in patients with untreated, EGFR-mutated, advanced non-small-cell lung cancer (RELAY): a randomised, double-blind, placebo-controlled, phase 3 trial. The Lancet Oncology.

[15] Zhang S, Cao M, Hou Z (2021). Angiotensin-converting enzyme inhibitors have adverse effects in anti-angiogenesis therapy for hepatocellular carcinoma. Cancer letters.

[16] Haddad RI, Schlumberger M, Wirth LJ (2017). Incidence and timing of common adverse events in Lenvatinib-treated patients from the SELECT trial and their association with survival outcomes. Endocrine.

[17] Liu J, Nicum S, Reichardt P (2018). Assessment and management of diarrhea following VEGF receptor TKI treatment in patients with ovarian cancer. Gynecol Oncol.

[18] Pal SK, Li SM, Wu X (2015). Stool Bacteriomic Profiling in Patients with Metastatic Renal Cell Carcinoma Receiving Vascular Endothelial Growth Factor-Tyrosine Kinase Inhibitors. Clin Cancer Res.

[19] Ianiro G, Rossi E, Thomas AM (2020). Faecal microbiota transplantation for the treatment of diarrhoea induced by tyrosine-kinase inhibitors in patients with metastatic renal cell carcinoma. Nat Commun.

[20] McQuade JL, Daniel CR, Helmink BA (2019). Modulating the microbiome to improve therapeutic response in cancer. Lancet Oncol.

[21] Schwabe RF, Greten TF (2020). Gut microbiome in HCC - Mechanisms, diagnosis and therapy. J Hepatol.

[22] Hahn AW, Froerer C, VanAlstine S (2018). Targeting Bacteroides in Stool Microbiome and Response to Treatment With First-Line VEGF Tyrosine Kinase Inhibitors in Metastatic Renal-Cell Carcinoma. Clin Genitourin Cancer.

[23] Huo Z, Yu S, Hong S (2016). A systematic review and meta-analysis of the risk of diarrhea associated with vandetanib treatment in carcinoma patients. OncoTargets and therapy.

[24] Schmidinger M, Danesi R (2018). Management of Adverse Events Associated with Cabozantinib Therapy in Renal Cell Carcinoma. Oncologist.

[25] Que Y, Liang Y, Zhao J (2018). Treatment-related adverse effects with pazopanib, sorafenib and sunitinib in patients with advanced soft tissue sarcoma: a pooled analysis. Cancer Manag Res.

[26] Yin X, Yin Y, Shen C (2018). Adverse events risk associated with regorafenib in the treatment of advanced solid tumors: meta-analysis of randomized controlled trials. OncoTargets and therapy.

[27] Secombe KR, Van Sebille YZA, Mayo BJ (2020). Diarrhea Induced by Small Molecule Tyrosine Kinase Inhibitors Compared With Chemotherapy: Potential Role of the Microbiome. Integr Cancer Ther.

