# Supplementary material for: Drug-related adverse events potentially predict the efficacy of apatinib on advanced hepatocellular carcinoma
Source: BMC Gastroenterol. 2022 Oct 31;22:441. doi: 10.1186/s12876-022-02542-0 (PMC9620633; doi:10.1186/s12876-022-02542-0)
Supplement: Supplementary file 1 — Additional file 1: Table S1. Baseline characteristics of patients before and after PSM (Non-HFS vs. HFS). Table S2. Baseline characteristics of patients before and after PSM (Non-Proteinuria vs. Proteinuria). Table S3. Baseline characteristics of patients before and after PSM (Non-diarrhea vs. Diarrhea) [file 12876_2022_2542_MOESM1_ESM.pdf]

Drug Safety

Drug-Related Adverse Events Predict the Efficacy of Apatinib on Advanced Hepatocellular Carcinoma

Xiaoying Gu[1]; Su Zhang[1]; Xuejiao Yang[1,2]; Tao Guan[1]; Zhenyu Hou[1]; Manqing Cao[3]; Huikai Li[1]; Ti Zhang[1,4]

[1] Department of Hepatobiliary Surgery, Tianjin Medical University Cancer Institute & Hospital, National Clinical Research Center for Cancer, Tianjin's Clinical Research Center for Cancer, Key Laboratory of Cancer Prevention and Therapy, Tianjin 300060, China

[2] Department of Anesthesiology, The Central Hospital of Enshi Tujia and Miao Autonomous Prefecture, Enshi 445000, Hubei, China. (Present address)

[3] Department of Breast Surgery, Tianjin Medical University Cancer Institute & Hospital, National Clinical Research Center for Cancer, Tianjin's Clinical Research Center for Cancer, Key Laboratory of Cancer Prevention and Therapy, Tianjin 300060, China.

[4] Department of Hepatic Surgery, Fudan University Shanghai Cancer Center, Shanghai Medical College, Fudan University, Shanghai, 200032, PR China. (Present address)

Corresponding authors:

Ti Zhang

Tel +86-21-6417-5590

Fax +86-21-6417-5590

Email zhangti@shca.org.cn

HuiKai Li

Tel +86-22-2335-9984

Fax +86-22-2335-9984

E-mail tjchlhk@126.com

Table S1 Baseline characteristics of patients before and after PSM (Non-HFS vs. HFS) **4**

Table S2 Baseline characteristics of patients before and after PSM (Non-Proteinuria vs. Proteinuria) .**5**

Table S3 Baseline characteristics of patients before and after PSM (Non-diarrhea vs. Diarrhea) **6**

Table S1 Baseline characteristics of patients before and after PSM (Non-HFS vs. HFS)

	**Before PSM**			**After PSM**		
	Non-HFS (N=126)	***HFS (N=81)***	**P value**	***Non-HFS (N=75)***	***HFS (N=75)***	**P value**
Sex			0.849			0.817
Female	18 (14.3%)	10 (12.3%)		12 (16.0%)	10 (13.3%)	
Male	108 (85.7%)	71 (87.7%)		63 (84.0%)	65 (86.7%)	
Age			0.610			0.867
< 60 years	72 (57.1%)	50 (61.7%)		47 (62.7%)	45 (60.0%)	
≥60 years	54 (42.9%)	31 (38.3%)		28 (37.3%)	30 (40.0%)	
Initial dose			0.011			1.000
500mg/d	4 (3.17%)	11 (13.6%)		4(5.3%)	5(6.7%)	
250mg/d	122 (96.8%)	70 (86.4%)		71(94.7%)	70(93.3%)	
ECOGPS			1.000			0.870
1	66 (52.4%)	42 (51.9%)		42 (56.0%)	40 (53.3%)	
0	60 (47.6%)	39 (48.1%)		33 (44.0%)	35 (46.7%)	
AFP			0.886			0.743
< 400μg/L	69 (54.8%)	46 (56.8%)		39 (52.0%)	42 (56.0%)	
≥400μg/L	57 (45.2%)	35 (43.2%)		36 (48.0%)	33 (44.0%)	
Child-Pugh			0.514			1.000
A	90 (71.4%)	62 (76.5%)		56 (74.7%)	57 (76.0%)	
B	36 (28.6%)	19 (23.5%)		19 (25.3%)	18 (24.0%)	
BCLC			1.000			1.000
B	25 (19.8%)	16 (19.8%)		15 (20.0%)	15 (20.0%)	
C	101 (80.2%)	65 (80.2%)		60 (80.0%)	60 (80.0%)	
Hepatitis			1.000			1.000
Hepatitis B	97 (77.0%)	63 (77.8%)		59 (78.7%)	59 (78.7%)	
Hepatitis C	4 (3.17%)	2 (2.47%)		3 (4.00%)	2 (2.67%)	
None	23 (18.3%)	15 (18.5%)		13 (17.3%)	13 (17.3%)	
TACE			0.013			0.824
no	41 (32.5%)	13 (16.0%)		11 (14.7%)	13 (17.3%)	
yes	85 (67.5%)	68 (84.0%)		64 (85.3%)	62 (82.7%)	
MVI			0.916			1.000
no	66 (52.4%)	41 (50.6%)		40 (53.3%)	40 (53.3%)	
yes	60 (47.6%)	40 (49.4%)		35 (46.7%)	35 (46.7%)	
EHS			0.847			1.000
no	59 (46.8%)	36 (44.4%)		33 (44.0%)	33 (44.0%)	
yes	67 (53.2%)	45 (55.6%)		42 (56.0%)	42 (56.0%)	

Abbreviations: ECOG PS, Eastern Cooperative Oncology Group performance status score; BCLC, Barcelona Clinic Liver Cancer; AFP, alpha-fetoprotein; MVI, macrovascular invasion; EHS, extrahepatic spread; HFS, Hand and foot syndrome.

Table S2 Baseline characteristics of patients before and after PSM (Non-Proteinuria vs. Proteinuria)

	**Before PSM**			**After PSM**		
	Non-Proteinuria (N=157)	***Proteinuria (N=50)***	**P value**	***Non-Proteinuria (N=46)***	***Proteinuria (N=46)***	**P value**
Sex			0.549			1.000
Female	23 (14.6%)	5 (10.0%)		6 (13.0%)	5 (10.9%)	
Male	134 (85.4%)	45 (90.0%)		40 (87.0%)	41 (89.1%)	
Age			0.317			1.000
< 60 years	89 (56.7%)	33 (66.0%)		31 (67.4%)	30 (65.2%)	
≥60 years	68 (43.3%)	17 (34.0%)		15 (32.6%)	16 (34.8%)	
Initial dose			0.011			1.000
500mg/d	7 (4.46%)	8 (16.0%)		5 (10.9%)	4 (8.7%)	
250mg/d	150 (95.5%)	42 (84.0%)		41 (89.1%)	42 (91.3%)	
ECOGPS			0.606			0.531
1	84 (53.5%)	24 (48.0%)		20 (43.5%)	24 (52.2%)	
0	73 (46.5%)	26 (52.0%)		26 (56.5%)	22 (47.8%)	
AFP			0.928			0.677
< 400μg/L	88 (56.1%)	27 (54.0%)		22 (47.8%)	25 (54.3%)	
≥400μg/L	69 (43.9%)	23 (46.0%)		24 (52.2%)	21 (45.7%)	
Child-Pugh			0.937			0.821
A	116 (73.9%)	36 (72.0%)		31 (67.4%)	33 (71.7%)	
B	41 (26.1%)	14 (28.0%)		15 (32.6%)	13 (28.3%)	
BCLC			0.869			1.000
B	32 (20.4%)	9 (18.0%)		7 (15.2%)	8 (17.4%)	
C	125 (79.6%)	41 (82.0%)		39 (84.8%)	38 (82.6%)	
Hepatitis			1.000			0.793
Hepatitis B	120 (76.4%)	40 (80.0%)		36 (78.3%)	37 (80.4%)	
Hepatitis C	5 (3.18%)	1 (2.00%)		0 (0.00%)	1 (2.17%)	
None	29 (18.5%)	9 (18.0%)		10 (21.7%)	8 (17.4%)	
TACE			0.590			1.000
no	39 (24.8%)	15 (30.0%)		14 (30.4%)	14 (30.4%)	
yes	118 (75.2%)	35 (70.0%)		32 (69.6%)	32 (69.6%)	
MVI			1.000			0.835
no	81 (51.6%)	26 (52.0%)		22 (47.8%)	24 (52.2%)	
yes	76 (48.4%)	24 (48.0%)		24 (52.2%)	22 (47.8%)	
EHS			0.405			0.835
no	69 (43.9%)	26 (52.0%)		21 (45.7%)	23 (50.0%)	
yes	88 (56.1%)	24 (48.0%)		25 (54.3%)	23 (50.0%)	

Abbreviations: ECOG PS, Eastern Cooperative Oncology Group performance status score; BCLC, Barcelona Clinic Liver Cancer; AFP, alphafetoprotein; MVI, macrovascular invasion; EHS, extrahepatic spread.

Table S3 Baseline characteristics of patients before and after PSM (Non-diarrhea vs. Diarrhea)

	**Before PSM**			**After PSM**		
	Non-diarrhea (N=160)	***Diarrhea (N=47)***	***P value***	***Non-diarrhea (N=43)***	***Diarrhea (N=43)***	***P value***
Sex			0.368			1.000
Female	24 (15.0%)	4 (8.51%)		4 (9.30%)	4 (9.30%)	
Male	136 (85.0%)	43 (91.5%)		39 (90.7%)	39 (90.7%)	
Age			0.200			1.000
< 60 years	90 (56.2%)	32 (68.1%)		29 (67.4%)	28 (65.1%)	
≥60 years	70 (43.8%)	15 (31.9%)		14 (32.6%)	15 (34.9%)	
Initial dose			0.048			1.000
500mg/d	8 (5.00%)	7 (14.9%)		3 (6.98%)	3 (6.98%)	
250mg/d	152 (95.0%)	40 (85.1%)		40 (93.0%)	40 (93.0%)	
ECOGPS			0.502			0.188
1	86 (53.8%)	22 (46.8%)		14 (32.6%)	21 (48.8%)	
0	74 (46.2%)	25 (53.2%)		29 (67.4%)	22 (51.2%)	
AFP			0.897			0.195
< 400μg/L	88 (55.0%)	27 (57.4%)		19 (44.2%)	26 (60.5%)	
≥400μg/L	72 (45.0%)	20 (42.6%)		24 (55.8%)	17 (39.5%)	
Child-Pugh			0.711			1.000
A	116 (72.5%)	36 (76.6%)		31 (72.1%)	32 (74.4%)	
B	44 (27.5%)	11 (23.4%)		12 (27.9%)	11 (25.6%)	
BCLC			1.000			1.000
B	32 (20.0%)	9 (19.1%)		9 (20.9%)	9 (20.9%)	
C	128 (80.0%)	38 (80.9%)		34 (79.1%)	34 (79.1%)	
Hepatitis			0.364			0.463
Hepatitis B	119 (74.4%)	41 (87.2%)		34 (79.1%)	37 (86.0%)	
Hepatitis C	5 (3.12%)	1 (2.13%)		0 (0.00%)	1 (2.33%)	
None	33 (20.6%)	5 (10.6%)		8 (18.6%)	5 (11.6%)	
TACE			0.297			1.000
no	45 (28.1%)	9 (19.1%)		8 (18.6%)	9 (20.9%)	
yes	115 (71.9%)	38 (80.9%)		35 (81.4%)	34 (79.1%)	
MVI			0.792			1.000
no	84 (52.5%)	23 (48.9%)		22 (51.2%)	22 (51.2%)	
yes	76 (47.5%)	24 (51.1%)		21 (48.8%)	21 (48.8%)	
EHS			0.757			0.829
no	72 (45.0%)	23 (48.9%)		20 (46.5%)	22 (51.2%)	
yes	88 (55.0%)	24 (51.1%)		23 (53.5%)	21 (48.8%)	

Abbreviations: ECOG PS, Eastern Cooperative Oncology Group performance status score; BCLC, Barcelona Clinic Liver Cancer; AFP, alphafetoprotein; MVI, macrovascular invasion; EHS, extrahepatic spread.
